# Effect of heat stress on blood-brain barrier integrity in iPS cell-derived microvascular endothelial cell models

**DOI:** 10.1371/journal.pone.0222113

**Published:** 2019-09-04

**Authors:** Tomoko Yamaguchi, Kentaro Shimizu, Yasuhiro Kokubu, Misae Nishijima, Shuko Takeda, Hiroshi Ogura, Kenji Kawabata

**Affiliations:** 1 Laboratory of Stem Cell Regulation, National Institutes of Biomedical Innovation, Health and Nutrition, Osaka, Japan; 2 Department of Traumatology and Acute Critical Medicine, Graduate School of Medicine, Osaka University, Osaka, Japan; 3 Department of Clinical Gene Therapy, Graduate School of Medicine, Osaka University, Osaka, Japan; 4 Laboratory of Biomedical Innovation, Graduate School of Pharmaceutical Sciences, Osaka University, Osaka, Japan; Emory University School of Medicine, UNITED STATES

## Abstract

The incidence of heatstroke has been increasing. Heatstroke has been shown to affect physiological barrier functions. However, there are few studies of the effect of heat stress on the blood-brain barrier (BBB) function. In this study, we investigated the influence of heat stress on brain microvascular endothelial cells *in vivo* and *in vitro*. Heatstroke model mice administered Texas Red-dextran showed leakage outside the brain vessel walls. In addition, trans-endothelial electrical resistance (TEER) value was significantly reduced in induced pluripotent stem (iPS) cell-derived brain microvascular endothelial cells under heat stress by reducing claudin-5 expression. In addition, our results showed that the expression level of P-glycoprotein (P-gp) was increased in iPS cell-derived brain microvascular endothelial cells under heat stress. Furthermore, serum from heatstroke model mice could impair the BBB integrity of iPS cell-derived brain microvascular endothelial cells. These results suggest that BBB integrity was affected by heat stress *in vivo* and *in vitro* and provide important insights into the development of new therapeutic strategies for heatstroke patients.

## Introduction

The blood-brain barrier (BBB) consists of brain microvascular endothelial cells that are surrounded and supported by astrocytes and pericytes. It plays critical roles in brain homeostasis and neural function by regulating the transfer of substances from the peripheral circulation into the brain. Brain microvascular endothelial cells show high trans-endothelial electrical resistance (TEER) and express selective influx and efflux transporters, for the import and export of molecules, respectively. BBB dysfunction is associated with many neurological diseases, such as multiple sclerosis and traumatic brain injury [1–3].

Heatstroke is characterized by an elevated core body temperature above 40°C, which leads to organ damage [4]. Notably, it is well known that heat stress impairs intestinal barrier integrity by increasing intestinal permeability and reducing epithelial resistance [5, 6]. It is also known that central nervous system (CNS) dysfunctions, including delirium, seizures, and coma, are symptoms of heatstroke. Heat stress could affect the function of the neurovascular unit (NVU) component cells, such as astrocytes, neuron [7], and microvascular endothelial cells [8]. It was previously reported that hyperthermia could induce mild BBB leakage in animal models [8, 9]. However, the molecular mechanisms of BBB disruption induced by heat stress are unclear. In addition, there are few studies in human heatstroke models. Therefore, *in vitro* human models are considered to be useful for examining detailed analysis of the molecular mechanisms of BBB disruption by heatstroke. Lippmann *et al*. recently developed methods to differentiate into brain microvascular endothelial cells from human pluripotent stem cells, such as embryonic stem (ES) cells and induced pluripotent stem (iPS) cells [10]. These iPS cell-derived brain microvascular endothelial cells have functions similar to those of tissue-derived brain microvascular endothelial cells, including physiologic barrier functions and BBB-specific transporter expression [10, 11]. Thus, we constructed an *in vitro* heatstroke model by using iPS cell-derived brain microvascular endothelial cells.

In this study, using the iPS cell-based model, we investigated the effect of heat stress on brain microvascular endothelial cells. When Texas Red-dextran was administered to mice under heatstroke conditions, leakage outside the brain vessel wall was observed, suggesting that heat stress could impair BBB integrity *in vivo*. In addition, heat stress could induce BBB disruption in an *in vitro* model using iPS cell-derived microvascular endothelial cells. Furthermore, the TEER value in iPS cell-derived brain microvascular endothelial cells was significantly reduced when treated with serum from heatstroke model mice. Thus, our results showed that BBB integrity was affected by heat stress *in vivo* and *in vitro*.

## Materials and methods

### Ethics statement

This study was approved by the institutional ethical review board at National Institutes of Biomedical Innovation, Health and Nutrition (Permit Number: iPS-3-27). Experimental procedures were approved by the Animal Care and Use Committee of Osaka University Graduate School of Medicine (Permit Number: 26-084-006). All of the experimental procedures were performed following our institutional guidelines. All surgery was performed under anesthesia used medetomidine (0.3 mg/kg), midazolam (4.0 mg/kg), and butorphanol (5.0 mg/kg) and all efforts were made to minimize suffering.

### Texas Red-dextran leakage assay

C57BL/6 wild-type mice were purchased from Nihon SLC Inc., and all animals were maintained under specific pathogen-free conditions. The permeability of the BBB was analyzed by the Texas Red-dextran leakage assay. C57BL/6 mice were injected intravenously with 250 μg of Texas Red-dextran (M.W. 3,000, ThermoFisher Scientific) before exposure to heat stress. To evaluate the effect of acute heat stress, mice were exposed to either normal conditions (22.6°C, 27% humidity) or heatstroke conditions (40.1°C, 50% humidity) for 2 h. Cerebrum were paraformaldehyde fixed and cut using cryostat for histological analysis. Images of brain sections were obtained with a BZ-X700 microscope (KEYENCE).

### Primary cerebral capillary isolation and

Mice were exposed to either normal conditions (23.9 ± 1.8°C, 31 ± 5.7% humidity) or heatstroke conditions (40.2 ± 0.2°C, 52.7 ± 4.6% humidity) for 2 h. Then, the capillaries were extracted from the cortex of mice according to the published protocol [12].

### Immunocytochemistry

Isolated vessels were fixed in ice-cold methanol for 10 min at room temperature. After washing with PBS, the vessels were blocked with 10% normal goat serum (Wako) in PBS supplemented with 0.1% Triton-X at room temperature for 1 h. After blocking, the cells were stained with a mouse anti-claudin-5 antibody (diluted 1/50; ThermoFisher Scientific) and a rabbit anti-platelet endothelial cell adhesion molecular-1 (PECAM-1) antibody (diluted 1/50; ThermoFisher Scientific) at 4 ^o^C overnight. Then, the cells were washed with PBS and stained with an Alexafluor488-conjugated secondary antibody and an Alexafluor594-conjugated secondary antibody (diluted 1/500; ThermoFisher Scientific). After washing, the nuclei were labeled with 4’, 6-diamidino-2-phenylindol (DAPI; Sigma) for 10 min at room temperature. Images were obtained with a BZ-X700 microscope (KEYENCE).

### Cell cultures

The human iPS cell line iMR90-4 [13] was maintained in mTeSR1 medium (Stem Cell Technologies), which was changed daily, and was passaged with 0.5 mM EDTA in PBS. Differentiation was performed according to the protocol of Lippman *et al*.[10]. Briefly, iPS cells were dissociated into single cells with Accutase (Merk-Millipore). Then the single cell suspension was seeded on Matrigel (BD Biosciences) in mTeSR1 medium with Y27632 (Wako). After 24 h, Y27632 was withdrawn, and the cells were re-fed mTeSR1 medium every 24 h. The iPS cells were cultured for 3 days and then switched to unconditioned medium [DMEM/F12 (ThermoFisher Scientific) with 20% Knock Out Serum Replacement (ThermoFisher Scientific), 1 x non-essential amino acid (NEAA: ThermoFisher Scientific), 1 mM Gluta-MAX (ThermoFisher Scientific), and 0.1 mM ß-mercaptoethanol (NACALAI TESQUE)]. Cells were re-fed unconditioned medium every 24 h for 6 days, and then switched to Human Endothelial-SFM (hESFM, ThermoFisher Scientific) supplemented with 1% human platelet poor plasma derived serum (PDS; Sigma) and 20 ng/mL fibroblast growth factor 2 (FGF2: Katayama Kagaku) and incubated for 2 days. Cells were dissociated with Accutase and plated on either 24-well transwell polyester membranes with 0.4 μm pores (BD Bioscience) or a tissue culture plate coated with a solution of fibronectin and type IV collagen (PharmaCo-Cell). Cells were subcultured in hESFM supplemented with 1% PDS and bFGF for 24 h and then the culture medium was changed to hESFM with 1% PDS. The Medium was not changed thereafter. To mimic heat stress conditions, the iPS cell-derived microvascular endothelial cells on the Transwell membranes were incubated at 42°C for 12 h. All experiments were initiated on day 10, because the value of trans-endothelial electrical resistance (TEER) was peaked on day 10. It has already been confirmed that differentiated cells in this method have the characteristics similar to brain microvascular endothelial cells [14].

### Measurement of TEER

The TEER values of the human iPS cell-derived brain microvascular endothelial cells incubated on the 24-well Transwell inserts were measured using Millicell ERS-2 (Merk-Millipore). To calculate the TEER (Ωxcm^2^ [the surface area of the insert, 0.3 cm^2^]), the resistance value of an empty filter coated with collagen IV and fibronectin was subtracted from each measurement.

### Permeability experiments

Before the transport studies, the medium was removed from the upper and lower chambers, and the chambers were washed with DPBS (Sigma) supplemented with HEPES (Sigma) and D-glucose (DPBS-H). DPBS-H containing 10 μg/mL sodium fluorescein (NaF, Sigma) was then loaded into the upper chamber, and DPBS-H was added to the lower chamber. After incubation for 30 min, the medium in the lower chamber was collected, and the NaF concentration in the sample was determined using a fluorescence multi-well plate reader (Genios, TECAN). The permeability coefficient was calculated as previously reported [15].

### Cell viability assay

Cells were cultured with 10% volume alamarBlue reagents (ThermoFisher Scientific). After incubation for 2 h, the medium was collected, and the cell viability was determined by an absorbance microplate reader (Sunrise, TECAN).

### Reverse transcription and quantitative polymerase chain reaction (RT-PCR)

Total RNA was isolated using RNAiso Plus reagent (Takara), and the cDNA was synthesized with the SuperScript VILO cDNA synthesis kit (ThermoFisher Scientific). The levels of claudin-5, occludin, Zonula occludens-1 (ZO-1), multidrug resistance protein 1 (MRP1, ABCC1), breast cancer resistance protein (BCRP, ABCG2), and P-glycoprotein (P-gp, ABCB1) mRNA were quantified using the SYBR Green detection system (Applied-Biosystems). The results were normalized to the housekeeping gene, glyceraldehyde 3-phosphate dehydrogenase (*GAPDH*). The sequences of the primers used in this study are listed in Table 1.

**Table 1:** Primer list used in quantitative real-time PCR.

Gene Name	(5') Forward Primers (3')	(5') Reverse Primers (3')
GAPDH	GGTGGTCTCCTCTGACTTCAACGT	GTGGTCGTTGAGGGCAATG
CLDN5	GTTCGTTGCGCTCTTCGTGA	GCTCGTACTTCTGCGACACG
OCL	CAGCAGCGGTGGTAACTTTG	TCCCTGATCCAGTCCTCCTC
ZO-1	TGATCATTCCAGGCACTCG	CTCTTCATCTCTACTCCGGAGACT
MRP1	CAAGGTGGATGCGAATGAGG	TGAGGAAGTAGGGCCCAAAG
BCRP	CTCTTCGGCTTGCAACAACT	TTCTCCTCCAGACACACCAC
P-gp	CACCCGACTTACAGATGATG	GTTGCCATTGACTGAAAGAA

### Western blot analysis

Cells were washed three times with sterile PBS, and cell lysates were obtained using radioimmunoprecipitation assay (RIPA) buffer (ThermoFisher Scientific) plus protease inhibitor cocktail (Roche). The proteins in the lysates were separated by SDS-PAGE on a 5–20% polyacrylamide gel (Wako) and were then transferred to polyvinylidene fluoride membranes (Merk-Millipore). After blocking with 5% skim milk in TBS containing 0.1% Tween 20 at room temperature for 1 h, the membranes were incubated with a mouse anti-claudin-5 antibody (diluted 1/250; ThermoFisher Scientific), a mouse anti-P-gp antibody (diluted 1/250; GeneTex), a rabbit anti-CD31 antibody (diluted 1/1000; abcam), or a mouse anti-ß-actin antibody (diluted 1/5000; Sigma-Aldrich) at 4 ^o^C overnight, and then with a HRP-conjugated anti-mouse IgG (Cell Signaling Technology) or a HRP-conjugated anti-rabbit IgG (Cell Signaling Technology) at room temperature for 1 h. The bands were detected with ECL Plus western blotting detection reagents (ThermoFisher Scientific), and the signals were visualized with a LAS-4000 imaging system (Fuji Film).

### Treatment with serum from heatstroke model mice

Blood samples were collected from the inferior vena cava after exposure to either normal conditions (22.6°C, 27% humidity) or heatstroke conditions (40.1°C, 50% humidity) for 2 h, and the mice were sacrificed. Blood samples on ice for 2–3 h, then it was centrifuged, and the serum was collected. iPS cell-derived microvascular endothelial cells on Transwell membranes were treated with 10% serum from normal or heatstroke model mice in hESFM supplemented with 1% PDS and bFGF. After incubation for 24 h, the TEER values of the human iPS cells-derived brain microvascular endothelial cells on 24-well Transwell inserts were measured using Millicell ERS-2.

### Statistical analysis

Statistical analyses were performed using an unpaired two-tailed Student’s t-test.

## Results

### The BBB was disrupted in heatstroke model mice

**Fig 1:**
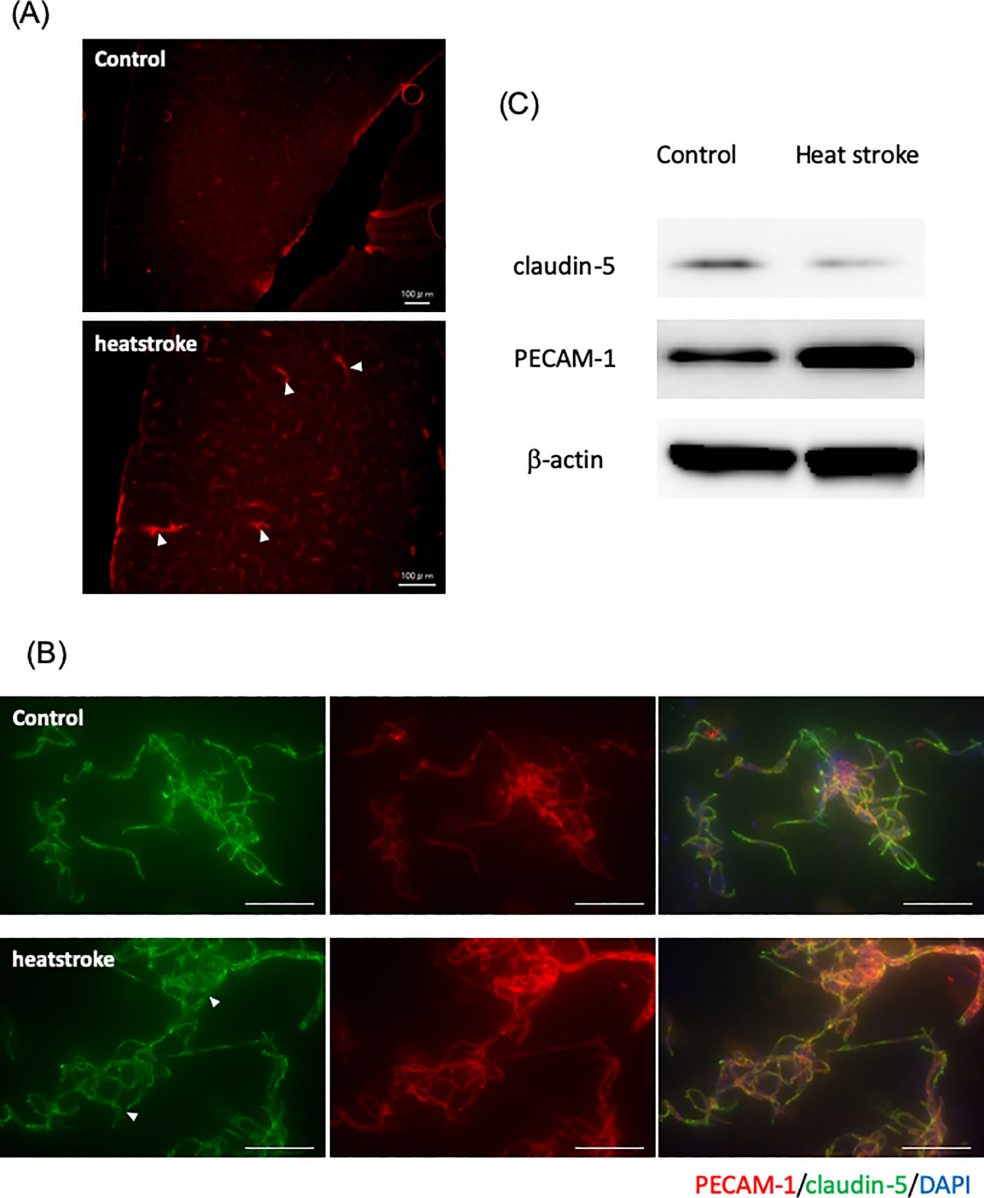
Heatstroke model mice show severe BBB leakage. (A) Vascular permeability, as measured by the leakage of Texas Red-dextran into the brain parenchyma (white arrows), was analyzed in brain tissue sections from normal mice (left) and heatstroke model mice (right). One representative image from five independent experiments is shown. Scale bar = 100 μm. (B) Expression of claudin-5 (Green) in brain microvascular endothelial cells (PECAM-1, Red) from normal mice (left) and heatstroke model mice (right) was evaluated by immunocytochemistry. One representative image from five independent experiments is shown. Scale bar = 100 μm. (C) Expression levels of claudin-5 and PECMA1 in brain microvessels from normal mice (n = 5) and heatstroke model mice (n = 5) were evaluated by western blotting. One representative image from two independent experiments is shown.

First, we evaluated the degree of BBB disruption in the heatstroke model mice. When Texas Red-dextran was intravenously administered to mice housed at normal temperature, it stayed within the circulation and did not leak into the brain (Fig 1A left). In contrast, when Texas Red-dextran was administrated to heatstroke model mice, leakage was evident outside the brain vessel wall (Fig 1A right). It is known that claudin-5 plays an important role in the barrier function of brain microvascular endothelial cells [16]. Therefore, we examined the expression of claudin-5 under heatstroke conditions. Our results showed that the expression level of claudin-5 protein was lower in the brain microvessels of heatstroke model mice than in normal mice (Fig 1B and 1C). In addition, we found that the expression level of PECAM-1 was increased in the heatstroke brain microvessels when compared to that level in normal brain microvessels (Fig 1B and 1C). These results showed that heat stress could impair BBB integrity by decreasing the expression of claudin-5.

### Heat stress could regulate the expression of tight junction-related genes

**Fig 2:**
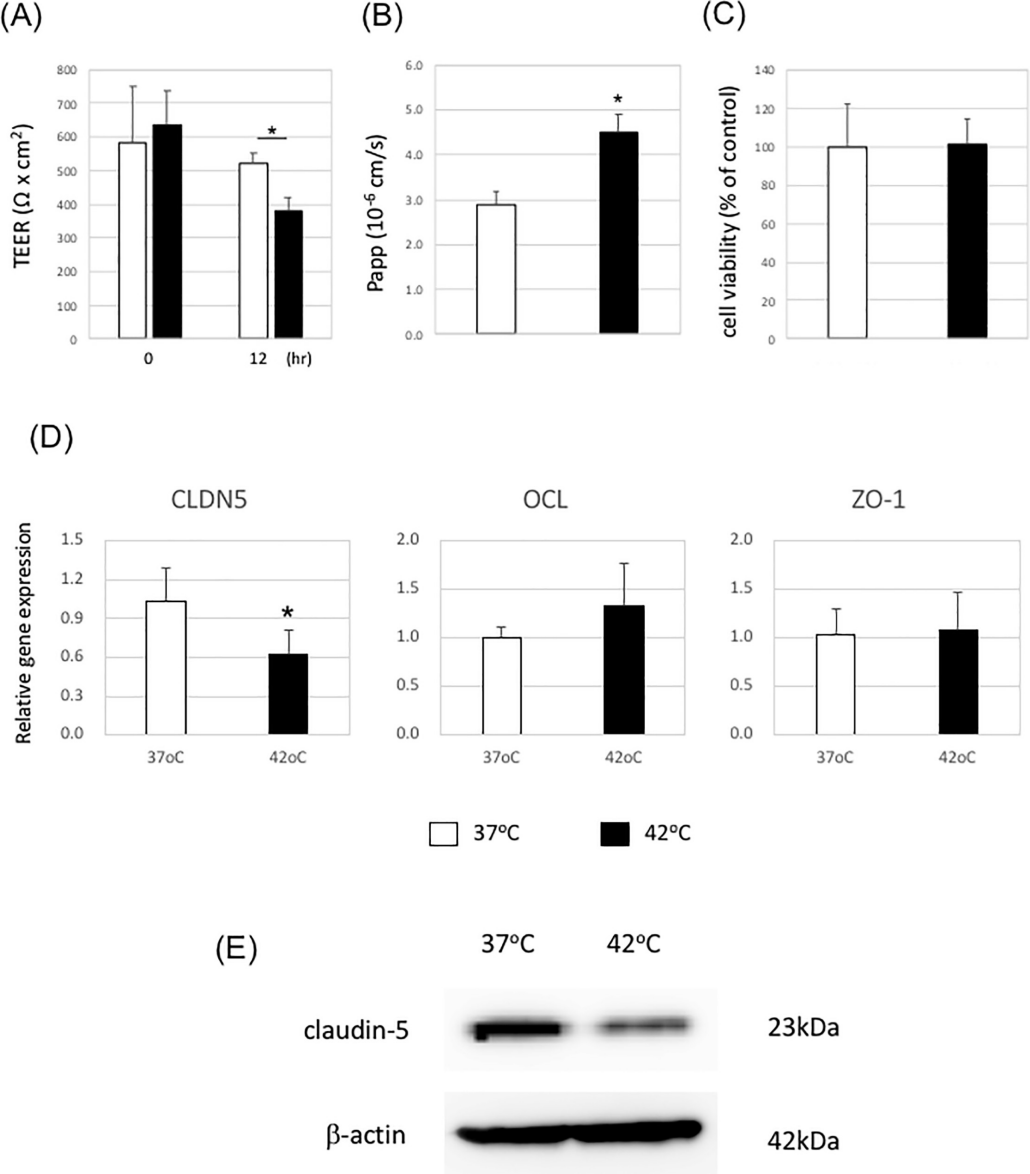
The barrier function of iPS cell-derived brain microvascular endothelial cells is disrupted under heat stress. iPS cell-derived brain microvascular endothelial cells on Transwell membranes were incubated at 37°C or 42°C for 12 h. (A) TEER values were monitored before and after heat stress exposure. Data are represented as the mean +/- S.D. from three independent experiments (n = 3). *P<0.05 (two-tailed Student’s t-test; compared with 37°C). (B) NaF flux across the cell monolayer was measured. Data are the mean +/- S.D. from three independent experiments (n = 3). *P<0.05 (two-tailed Student’s t-test; compared with 37°C). (C) Cell viability was measured by alamarBlue. Data are the mean +/- S.D. from three independent experiments (n = 3). *P<0.05 (two-tailed Student’s t-test; compared with 37°C). (D) Gene expression levels of claudin-5, occludin, and ZO-1 were measured by quantitative RT-PCR. Gene expression levels of cells at 37°C were set to 1.0. Data are the mean +/- S.D. from three independent experiments (n = 3). *P<0.05 (two-tailed Student’s t-test; compared with 37°C). (E) Expression levels of claudin-5 were evaluated by western blotting. One representative image from three independent experiments is shown.

Next, we examined BBB permeability under heat stress using iPS cell-derived brain microvascular endothelial cells. *Lippmann et al*. previously established a method for the differentiation of brain microvascular endothelial cells from human iPS cells [10, 11]. These iPS cell-derived brain microvascular endothelial cells show tight junction formation, which is a characteristic of brain microvascular endothelial cells. First, the influence of heat stress on BBB integrity and paracellular permeability were evaluated by measuring the TEER and sodium fluorescein (NaF) flux. When iPS cell-derived brain microvascular endothelial cells were exposed to a high temperature, the heat stress significantly reduced the TEER value compared with that in cells incubated under normal temperature conditions (Fig 2A). In addition, the paracellular permeability, as measured by NaF flux, was significantly increased after heat stress (Fig 2B). These results demonstrate the correlation between the formation of tight junctions and paracellular permeability. Next, we investigated the viability of iPS cell-derived brain microvascular endothelial cells under heat stress, and no reduction in cell viability was observed under heat stress (Fig 2C). These results showed that heat stress could induce the BBB disruption in iPS cell-derived brain microvascular endothelial cells.

Because the paracellular permeability of microvascular endothelial cells is regulated by tight junctions, we analyzed the expression of the tight junction-related genes, including claudin-5, occludin, and ZO-1, in iPS cell-derived brain microvascular endothelial cells. The mRNA expression levels of occludin and ZO-1 were unchanged under heat stress (Fig 2D). In contrast, the mRNA expression level of claudin-5 was lower under heat stress than under normal conditions (Fig 2D). We also compared the expression levels of claudin-5 protein in iPS cell-derived brain microvascular endothelial cells under heat stress and normal temperature conditions by western blot analysis. Our results showed that the claudin-5 protein level was decreased under heat stress (Fig 2E). This result was consistent with the quantitative RT-PCR results. These results suggest that heat stress could induce the BBB disruption by reducing claudin-5 expression.

### Heat stress could regulate the expression of P-gp

**Fig 3:**
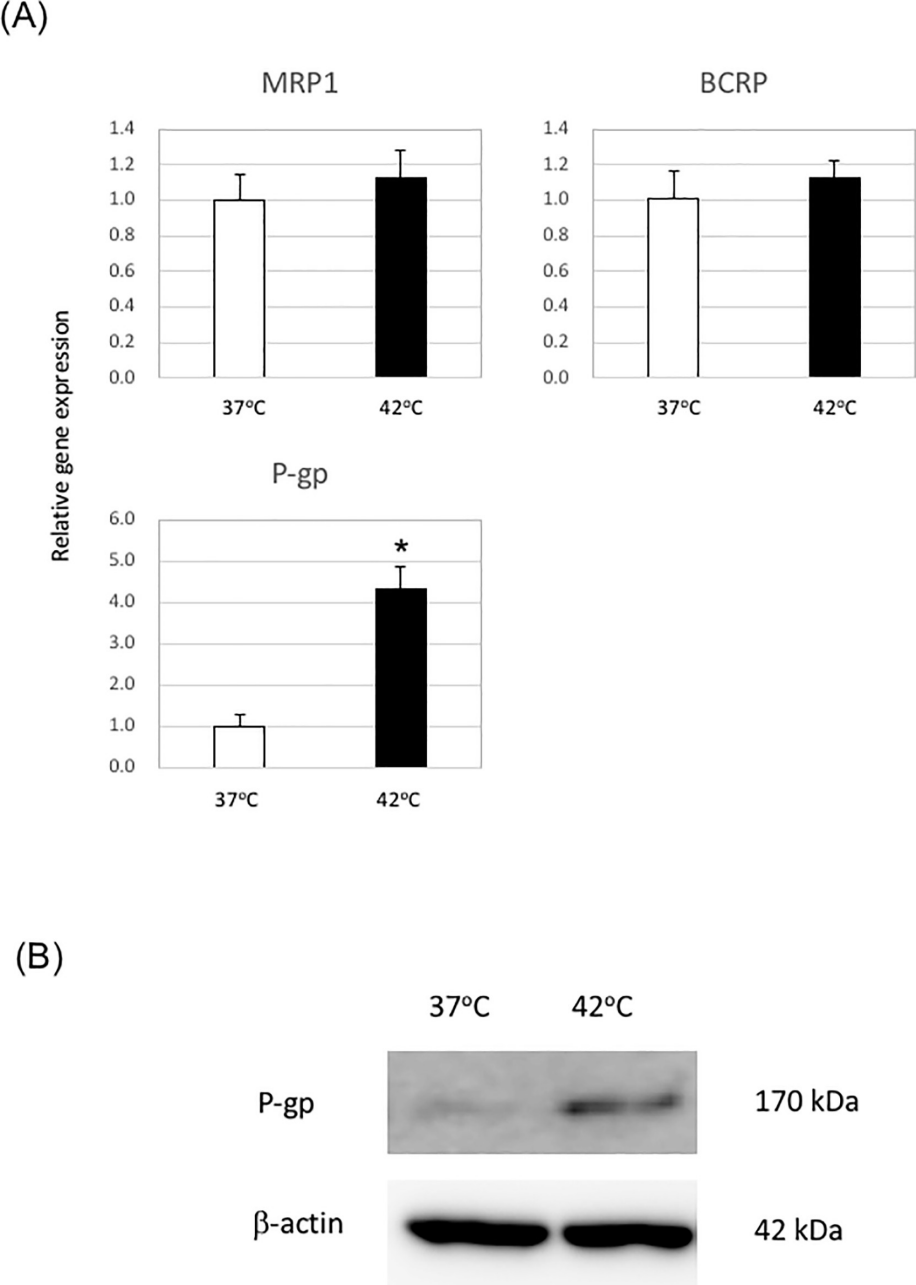
Expression of P-gp in iPS cell-derived brain microvascular endothelial cells is regulated by heat stress. (A) iPS cell-derived microvascular endothelial cells on Transwell membranes were incubated at 37°C or 42°C for 12 h, and then the gene expression levels of MRP-1, BCRP, and P-gp were measured by quantitative RT-PCR. Gene expression levels at 37°C were set at 1.0. Data are the mean +/- S.D. from three independent experiments (n = 3). *P<0.05 (two-tailed Student’s t-test; compared with 37°C). (B) Expression levels of P-gp were evaluated by western blotting. One representative image from three independent experiments is shown.

To protect the brain from exposure to unnecessary metabolites and drugs and to provide nutrients to the brain, various efflux and influx transporters are expressed in brain microvascular endothelial cells [17]. We next analyzed the expression of various influx/efflux transporters after heat stress. The mRNA expression levels of MRP1 and BCRP were unchanged under heat stress (Fig 3A). In contrast, the mRNA expression level of P-gp was significantly increased in iPS cell-derived brain microvascular endothelial cells under heat stress (Fig 3A). We also compared the expression levels of P-gp protein in iPS cell-derived brain microvascular endothelial cells under heat stress and normal temperature conditions by western blot analysis. Our results showed that the P-gp protein level was increased in iPS cell-derived brain microvascular endothelial cells under heat stress (Fig 3B). This result was consistent with the quantitative RT-PCR analysis. These results suggest that heat stress could regulate the expression of P-gp.

### Serum from a mouse model of heatstroke could influence of BBB permeability

**Fig 4:**
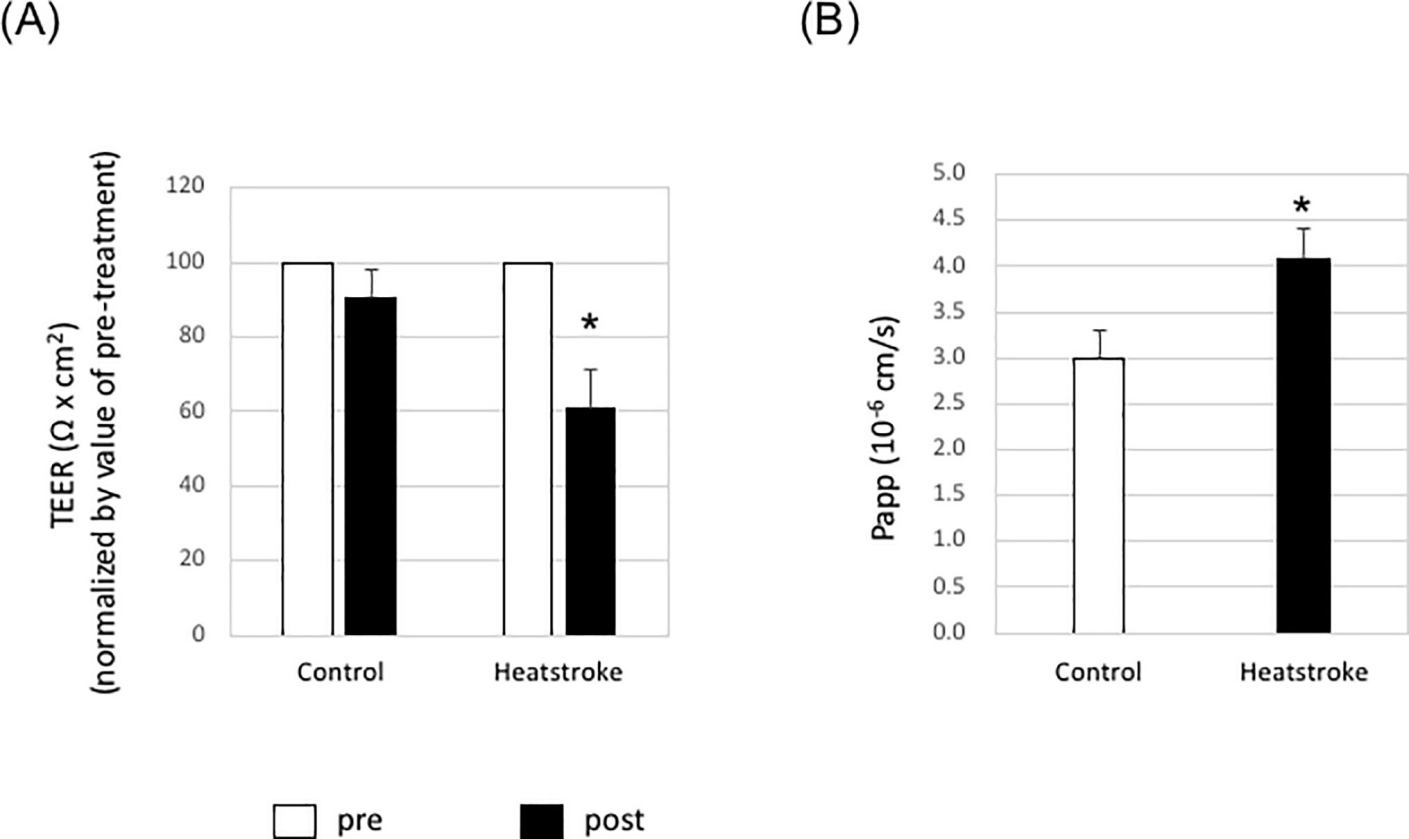
Barrier function of iPS cell-derived brain microvascular endothelial cells was disrupted by serum from heat stroke model mice. iPS cell-derived microvascular endothelial cells on Transwell membranes were treated with serum from normal mice (n = 5) or heatstroke model mice (n = 5). (A) TEER values were monitored before (used as a baseline) and after treatment with serum from normal or heatstroke model mice. The TEER values at pre-treatment were set at 100%. Data are the mean +/- S.D. from three independent experiments. *P<0.05 (two-tailed Student’s t-test; compared with the Control). (B) Permeability of NaF across the cell monolayer was measured. Data are the mean +/- S.D. from three independent experiments. *P<0.05 (two-tailed Student’s t-test; compared with the Control).

In Figs 2 and 3, we examined the direct effects of heat stress on iPS cell-derived brain microvascular endothelial cells. However, it has been reported that some organs besides the brain are damaged by heat stress [4]. It is possible that effector molecules, such as inflammatory cytokines, are released into the circulation from these damaged organs. Therefore, we next examined the influence of serum from heatstroke model mice on the formation of tight junctions by iPS cell-derived brain microvascular endothelial cells. The results showed that the TEER value was significantly reduced in iPS cell-derived brain microvascular endothelial cells treated with serum from heatstroke model mice (Fig 4A). In addition, paracellular permeability, as measured by NaF flux, was significantly increased following treatment with serum from heatstroke model mice (Fig 4B). These results showed that some molecules present in the circulation of the heatstroke mouse model affect BBB integrity.

## Discussion

In the current study, we investigated the influence of heat stress on human brain microvascular endothelial cells, and we demonstrated for the first time, that heat stress could induce BBB disruption by reducing claudin-5 expression *in vivo* and *in vitro* using a mouse model and human iPS cell-derived brain microvascular endothelial cells. Previous studies have shown that hypoxia inducible factor-1α (HIF-1α could regulate the expression of tight junction-related genes, including claudin-5, in cerebral ischemia using iPS cell-derived brain microvascular endothelial cells [18], and heat stress is known to induce the expression of HIF-1α via heat shock proteins [19]. Therefore, in heatstroke models, it is also possible that tight junction-related genes are also regulated by HIF-1α. We found the upregulated expression of PECAM-1 in brain microvessels of heatstroke model mice, suggesting that upregulated expression of PECAM-1 would play some roles in the BBB impairment under heatstroke. In other possibilities, vascular endothelial growth factor (VEGF), which is induced in the endothelial cells under heat stress [20], might participate in the BBB disruption.

We also found that heat stress could induce the expression of P-gp. As previously published, heat stress-induced some molecules, such as HIF-1α [21] and cyclooxygenase-2 (COX-2) [22], could induce the expression of P-gp. Notably, P-gp is known to play an important role in the efflux of various chemical mediators. Thus, as a defense mechanism, it is possible that the upregulation of P-gp expression could protect the brain from the invasion of harmful substances from the peripheral circulation.

It was previously reported that lipopolysaccharide (LPS) and high-mobility group box 1 (HMGB1) were released from the gut lumen into the systemic circulation under heatstroke conditions [23]. HMGB1 [24] and LPS [25] could induce the disruption of the BBB. In addition, various types of inflammatory cytokines, including tumor necrosis factor-α (TNF-α) and IL-1ß, were contained in the serum of heatstroke model rats [26]. It was previously reported that claudin-5 is a common target of inflammatory mediators, including interleukin (IL)-1ß [27] and TNF-α [28], using *in vitro* BBB models. Therefore, it is thought that various types of inflammatory cytokines and/or pathogenic factors are present in the serum of heatstroke model mice. We are now engaged in an ongoing investigation of serum samples from human heatstroke patients. Further studies are needed to provide a detailed analysis of the molecular mechanisms underlying BBB disruption by serum from heatstroke patients.

In summary, we showed that heat stress could induce BBB disruption by reducing claudin-5 expression. We also found that the expression of P-gp, which is a multidrug efflux transporter, could be regulated by heat stress in iPS cell-derived brain microvascular endothelial cells. Furthermore, our results showed that molecules that could disrupt BBB integrity were present in serum from heatstroke model mice. These results showed that heat stress might directly and indirectly regulate BBB function.
